# Discovery of a new inland population of *Leptoconops noei* in Italy with sequencing of the first complete mitochondrial genome for the genus

**DOI:** 10.1111/mve.12828

**Published:** 2025-07-11

**Authors:** Giovanni Naro, Gianluca Lombardo, Alessandro Alvaro, Laura Soresinetti, Francesco Frati, Luigi Marvasi, Emanuele Montomoli, Claudio Bandi, Paolo Gabrieli, Carlo Polidori, Sara Epis

**Affiliations:** ^1^ Department of Biosciences and Pediatric Clinical Research Center “Romeo Ed Enrica Invernizzi” University of Milan Milan Italy; ^2^ Department of Life Sciences University of Siena Siena Italy; ^3^ National Biodiversity Future Center Palermo Italy; ^4^ GLP Research Center Farefarma‐Emozoo Siena Italy; ^5^ Department of Molecular and Developmental Medicine University of Siena Siena Italy; ^6^ VisMederi Siena Italy; ^7^ Department of Environmental Science and Policy (ESP) University of Milan Milan Italy

**Keywords:** bites, blood‐sucking midges, Ceratopogonidae, morphological identification, Tuscany

## Abstract

The Ceratopogonidae family, comprising over 6000 described species, includes the genus *Leptoconops*, which has been understudied despite its ecological significance and biting nuisance to humans. Here, we document the presence of inland *Leptoconops noei* populations for the first time in Italy in an area previously considered environmentally atypical for this species. Our findings expand the known range of *L. noei*, traditionally confined to coastal areas, and highlight its potential to colonise diverse habitats. Interestingly, despite the thorough morphological and molecular identification of sampled individuals and their clear assignment to the species *L. noei*, a notable morphological variability was observed in the sensory structures of the maxillary palps, suggesting the possibility of a new morphotype or environment‐driven adaptations. Phylogenetic analyses of *COX1* sequences revealed negligible genetic differentiation between the newly discovered inland population and coastal populations, suggesting recent separation or gene‐flow connection. These findings underscore the ecological adaptability of *L. noei*, which poses risks of human‐biting nuisance, allergic reactions and even possible economic losses in recreative areas, in a range of locations wider than previously assumed. Additionally, this study reports the first complete mitochondrial genome for the genus *Leptoconops*, offering valuable genetic insights for taxonomic clarification, evolutionary studies and future research on the biology of Ceratopogonidae.

## Graphical abstract

- The genus *Leptoconops* is poorly investigated, despite its ecological relevance and biting nuisance. The studies in Italy focused only on the coastal areas.
- Inland populations of *Leptoconops noei* are documented for the first time in Italy. We record their presence in an area covering Siena district, Tuscany region, in an atypical environment for the genus.
- A new morphological variant is described for *L. noei*. The complete mitochondrial genome is sequenced and annotated, remarkably being the first for the genus *Leptoconops*.

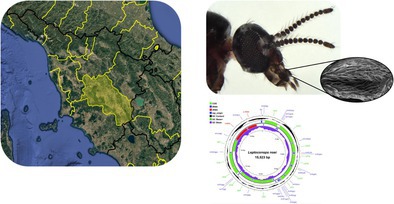

## INTRODUCTION

The Ceratopogonidae (Diptera: Nematocera) includes over 6000 described species (Borkent & Dominiak, 2020), though it is estimated that over 15,000 species have yet to be described (Marquardt & Kondratieff, 2005). Species of blood‐sucking midges are classified in four genera of Ceratopogonidae, namely *Austroconops* Wirth & Lee, *Culicoides* Latreille, *Forcipomyia* Lenz and *Leptoconops* Skuse (Kettle, 1977). Among these, the genus *Culicoides*, with over 1000 described species worldwide, is extensively studied due to its widespread and harmful nature, including vector competence for many arboviruses such as Bluetongue virus and Schmallenberg virus (Carpenter et al., 2013), both responsible for recent outbreaks in Europe affecting livestock, and also for viruses involved in human diseases like Oropouche Fever (Zhang et al., 2024). The other three genera, however, remain comparatively understudied.

The genus *Leptoconops*, definitively established in the early 20th century (Carter, 1921) and having a worldwide distribution with its 154 known species (Borkent & Dominiak, 2020), exemplifies this knowledge gap, despite having evolutionary, biological and parasitological relevance. First, this genus represents the ancestral branch of the family Ceratopogonidae (Beckenbach & Borkent, 2003), with the oldest fossil of the genus dating back to 120 MYA (Borkent, 2001). Second, these tiny biting midges tend to fly in large swarms and frequently attack humans. Indeed, by easily passing through clothing and mosquito nets due to their small size, they can cause wounds all over the body with their bites (González et al., 2013; Noè, 1907). This implies heavy nuisance for humans, as their bites can lead to discomfort, pain and occasionally severe allergic reactions (Cocchi et al., 1985; González et al., 2025). Furthermore, there is a poor understanding of their potential role as a vector of pathogens, even though female *Leptoconops* feed on the blood of vertebrates (Široký et al., 2007; Wirth & Atchley, 1973). Finally, nothing is known about the genomics and cytogenetics of the genus, except for sequences of barcoding genes of a few species (see Table S1).

Six out of the 11 European species of *Leptoconops* occur along the coasts of Italy, especially in central and southern regions of the country (Boorman et al., 1995; Clastrier, 1973; Cocchi, Menichetti, Vichi, Tamburro, & Gatti, 1986). The subgenus *Leptoconops* sensu stricto includes *L. bezzii* (Noé, 1905), *L. bidentatus* (Gutsevich, 1960), *L. irritans* (Noé, 1905) and *L. noei* (Clastrier & Coluzzi, 1973), while the subgenus *Holoconops* comprises *L. ccc* (Kieffer, 1908) and *L. gallicus* (Clastrier, 1973). Generally, their habitats are marshy and sandy coasts, river mouths and riverbanks. The life cycle of these midges requires sandy or clayey soil that is periodically submerged by water, as the larvae live in wet substrates, but the pupa needs a drier substrate to develop (Clastrier, 1972; Cocchi, Menichetti, Vichi, Tamburro, & Gatti, 1986; Rioux et al., 1968; Rioux & Descous, 1965). Tidal cycles, river flooding and stagnant water in swamps guarantee these conditions (Raspi et al., 2007). These habitats have been declining for decades, and the problems associated with *Leptoconops* are largely neglected. Notably, coastal Tuscany, particularly the Grosseto district, hosts all the aforementioned species. Indeed, most of the reports and investigations conducted on this genus focus on populations from this area, specifically within the ancient marshes, now in part reclaimed and cultivated, named Maremma (Belardinelli et al., 2010; Bettini et al., 1969a, 1969b, 1969c; Bettini & Finizio, 1968; Clastrier & Coluzzi, 1973; Cocchi et al., 1985; Cocchi, Menichetti, Vichi, & Tamburro, 1986; Cocchi, Menichetti, Vichi, Tamburro, & Gatti, 1986; Majori et al., 1970, 1971; Noè, 1907; Polidori et al., 2023; Raspi et al., 2007). The distribution of *Leptoconops* in Italy includes other sporadic records from coastal areas of the Regions of Lazio (Noè, 1907), Basilicata, Puglia (Carrieri et al., 2007; Moleas et al., 1998), Sardinia (Bettini et al., 1969a) and Sicily (Lavagnino et al., 1990).

The present study reveals the first records of these biting midges in inland areas of Italy, where their occurrence has remained completely undocumented until now. In particular, we report stable populations, as yet unrecorded, of *L. noei* in central Tuscany (Siena district) and highlight morphological differences with the previously described coastal populations. Furthermore, with the purpose of generating novel knowledge and tools for population evolutionary studies on these insects, we also present the first fully sequenced mitochondrial genome for the genus *Leptoconops*.

## MATERIALS AND METHODS

### Insect sampling in Siena district

**FIGURE 1:**
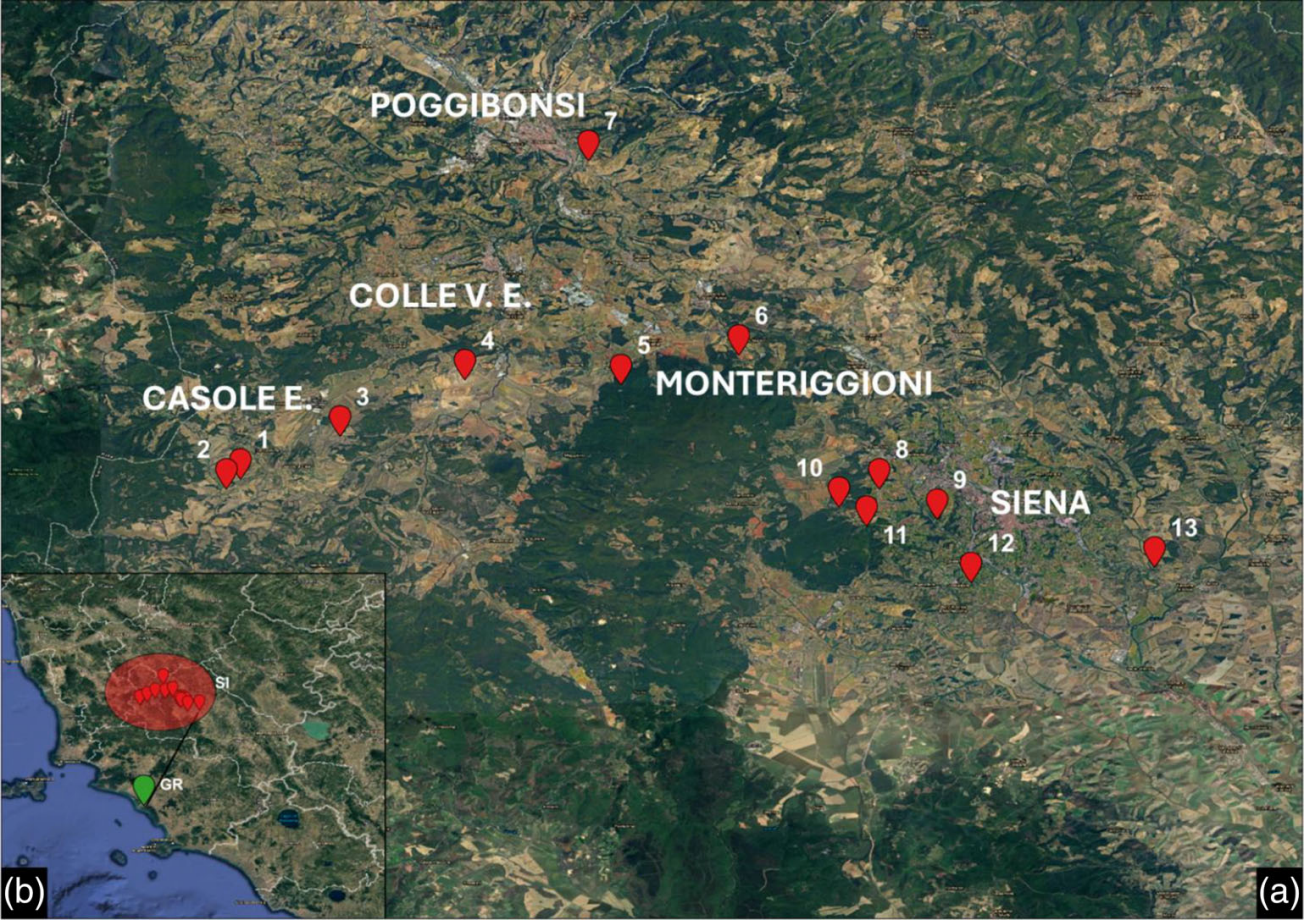
(a) Map of Siena district sites. (b) Tuscany region, distance between Siena sites and Grosseto site highlighted. Made with QGis.

Due to our increasing interest in investigating the genus *Leptoconops*, following a first study performed in coastal southern Tuscany (Polidori et al., 2023), we decided to extend our sampling activity in search of these midges in inland areas of this region. First, during the summer 2022 and 2023, 40 females of midges were serendipitously collected in the municipality of Casole d'Elsa (43°20′28″ N 11°02′54.6″ E), Siena district. Casole d'Elsa is located at an altitude of 417 m, on the slopes of the hills that characterise inland Tuscany (Site 1; Figure 1a). The mean temperatures are 23°C in summer and 5°C in winter, and annual precipitation has a maximum of 85 mm in November and a minimum of 20 mm in July (Consorzio LaMMA, 1991‐2020). The land use is dominated by agricultural activities and livestock, interspersed with deciduous woods. Then, we decided to heavily extend the sampling campaign during the summer 2024 not only in Casole d'Elsa but also in the surrounding Siena district.

The whole field collection was conducted in five municipalities, covering an area of 150 square kilometres: Casole d'Elsa, Colle di Val d'Elsa (43°25′21″ N 11°07′36″ E), Monteriggioni (43°22′01.15″ N 11°13′04.93″ E), Poggibonsi (43°28′ N 11°09′ E) and Siena (43°19′06″ N 11°19′53″ E) (see Figure 1). The adult midges were captured using a mechanical aspirator, catching the insects while they were resting on a black towel, which, we noticed, attracted them, or while they tried to bite the operators. The collected samples were immediately placed in a solution of 70% ethanol for later conservation at −20°C.

### Morphological identification

All the samples were identified at subgenus level and preliminary assigned to a species using a LEICA M50 stereomicroscope (Leica Microsystems, Wetzlar, Germany), according to the morphological keys provided by Carter (1921). Due to the limited and recent information about the identification and distribution of some species of *Leptoconops* in Italy, we performed further detailed observations to validate our preliminary key‐based identification.

A high‐precision digital stereomicroscope VHX‐7000 (Keyence, Osaka, Japan) was used for species identification based on the keys of Clastrier and Coluzzi (1973) and Clastrier (1972), and on a previous morphological analysis (Polidori et al., 2023). Three individuals from each site 1, 3, 4, 5, 7, 11, 12 were slide‐mounted and visualised under an optic microscope LEICA DM1000 (Leica Microsystems, Wetzlar, Germany). Two individuals from site 1 and two individuals of *L. noei* from Grosseto district were also selected to visualise morphological features under a scanning electron microscope (SEM) (Zeiss LEO 1430, Zeiss, Oberkochen, Germany). The samples were sputter‐coated with gold and mounted to aluminium stubs for SEM analysis. High vacuum conditions with a resolution of 3.0 nm at 30 kV (Secondary electrons [SE]), 10 nm at 3 kV (SE) and 4.0 nm at 30 kV (Backscattered electrons‐BSEs) were used. The accelerating voltage was 26 kV, the high vacuum was 53.3–66.6 Pa, and the working distance was 10 mm.

### *COX1* sequencing and phylogenetic analyses

Species identification was confirmed through molecular gene sequencing on specimens from different sites and years of collection: five from site 1 (one collected in 2022, two in 2023 and two in 2024), two from site 7 and two from site 13. DNA extraction and amplification of the mitochondrial *cytochrome c oxidase subunit 1* gene (*COX1*) were performed as described in Polidori et al. (2023). PCR products were sequenced by Eurofins Genomics (Ebersberg, Germany). Sequences were deposited in GeneBank (see Table S1).

As for the phylogenetic analyses, all *Leptoconops* sensu lato *COX1* entries in GenBank were downloaded and aligned for phylogenetic analysis. The length of the analysed sequences was 480 bp. Included entries were: 5 *Leptoconops noei*, 15 *Leptoconops irritans*, 11 *Leptoconops nipponensis*, 7 *Leptoconops* sp. and 3 *Leptoconops bezzii* (previously deposited by us but unpublished) together with five closely related sequences from outgroup species. To the aforementioned GenBank entries, we added 10 *Leptoconops noei* from Siena and 1 from Grosseto (Italy) and 1 *Leptoconops kerteszi* for a total of 59 *COX1* sequences. The Maximum likelihood tree was constructed in Mega12 (Kumar et al., 2024) using the general time reversable (GTR) substitution model and uniform rates for 1000 bootstraps. Information on all samples used for phylogenetic analysis and accession numbers can be found in Table S1. Average pairwise nucleotide diversity values were calculated using DNAsp (Rozas et al., 2017) according to the Jukes and Cantor correction.

### Mitochondrial genome sequencing

DNA extraction for mitochondrial genome sequencing was performed using the DNeasy Blood & Tissue kit (Qiagen, Hilden, Germany), following a revised protocol. Given the size of the midges, pools of 40 individuals of the *L. noei* species, collected from Siena district (Site 1; Figure 1a) or Grosseto district were used for DNA extraction. Samples of *L. noei* collected in June 2024 in Marina di Alberese (42°40′26″ N 11°02′51″ E; Figure 1b) in the Grosseto district, located 3 kilometres from the sea at an altitude of 1 metre above sea level, were also included in the mitochondrial genome sequencing to enable a comparison between the two genomes. The two preparations of DNA, concentrated at 60 ng/μl, were sent to Macrogen (Seoul, Korea) for Whole Genome Sequencing. Libraries were prepared using the TruSeq DNA PCR‐Free kit (350 paired‐end bp target) and run on the Illumina NovaSeqX.

Raw sequencing reads were prepared for mitochondrial DNA (mtDNA) analysis using a custom pipeline (modified from Lombardo et al., 2022) where *bcl* format files are demultiplexed and converted to *fastq* files using Illumina's proprietary C++ package bcl2fastq2 v.2.20 on a single node. The pipeline then recognises paired‐end read *fastq* files and trims adapters using TrimGalore v.0.6.10 (Krueger, 2015), pair‐end reads mode with Phred score threshold set to ‐q 30. Quality control is then performed with FastQC v.0.12.0 (Babraham Bioinformatics) and samples that pass the quality check are flagged for use. To obtain mitochondrial sequences from our whole genome data a novel reference mtDNA for *L. noei* had to be generated *de novo*. This was done by using a previously available *COX1* sequence from Genbank (OM672379, Polidori et al., 2023) and using it as a seed in SPAdes v.4.0.0 (Prjibelski et al., 2020) for the scaffolding. SPAdes was run with the following arguments: ‐careful, −only‐assembler ‐k (21, 33, 55, 77). To confirm the obtained scaffold, the mitogenome was also reconstructed in Geneious Prime® v.2025.0.3 (Kearse et al., 2012) using the built‐it mapper tool by once again starting from the published *COX1* accession and extending the sequence in both directions until repeating patterns emerged, indicating circularisation.

The obtained mtDNA was then aligned with *Culicoides arakawae* (NC_009809, Matsumoto et al., 2009) and *Forcipomyia makanensis* (MK000395, Jiang et al., 2019) to confirm the gene order, position and to establish ORFs. Once gene products were aligned and nuclear mitochondrial DNA (NUMTs) were confirmed not to be present, the sequence was used as a reference to re‐map our *fastq* files using an automated pipeline (modified from Broggini et al., 2024). This creates index files for the new reference sequence using bwa‐mem2 v.2.2.1 (Vasimuddin et al., 2019) and SAMtools v.1.2.1 (Danecek et al., 2021) and a sequence dictionary with Picard v.3.3.0 (https://broadinstitute.github.io/picard/). Previously flagged High Quality *fastq* files are then once again parsed through bwa‐mem2 (*fastq→sam*) and SAMtools (sam → bam) and mapped to the reference sequence. The mapped *bam* files were then converted to *vcf* files using GATK v.4.6.1.0 (Van der Auwera & O'Connor, 2020) with the HaplotypeCaller function. Finally, *vcf* files are converted to *fasta* using SAMtools' bcftools package. Files were then viewed in Geneious Prime®.

## RESULTS

### Morphology

A total of 563 *Leptoconops* females were collected across 13 sites in five municipalities. A detailed list of sites and samples collected is provided in Table 1. The sites where most individuals were collected were site 1 and site 2, located near a farm where we had free access, hosting cattle, horses and ducks. Sites 3, 7, 10, 11 and 13 were chosen for their proximity to farms with livestock and poultry, even though we did not have direct access to the properties. Sites 4, 5, 6, 8, 9 and 11 were also located in the countryside, near cultivated land and on the outskirts of the cities.

**TABLE 1:** Summary of the 2024 collection campaign. Site names, geographical information and number of specimens collected are reported.

Site	Coordinates	Municipality	*N*
1	43°20′30″ N 11°01′43″ E	Casole d'Elsa (SI, Italy)	325
2	43°20′16″ N 11°01′23″ E	Casole d'Elsa (SI, Italy)	94
3	43°21′31″ N 11°04′04″ E	Casole d'Elsa (SI, Italy)	18
4	43°22′51″ N 11°07′01″ E	Colle di Val d'Elsa (SI, Italy)	25
5	43°22′44″ N 11°10′42″ E	Monteriggioni (SI, Italy)	20
6	43°23′26″ N 11°13′29″ E	Monteriggioni (SI, Italy)	1
7	43°28′02″ N 11°09′56″ E	Poggibonsi (SI, Italy)	31
8	43°20′16″ N 11°16′48″ E	Siena (Italy)	10
9	43°19′33″ N 11°18′10″ E	Siena (Italy)	3
10	43°19′50″ N 11°15′51″ E	Siena (Italy)	8
11	43°19′23″ N 11°16′30″ E	Siena (Italy)	14
12	43°18′03″ N 11°18′58″ E	Siena (Italy)	2
13	43°18′25″ N 11°23′18″ E	Siena (Italy)	12
Total			563

**FIGURE 2:**
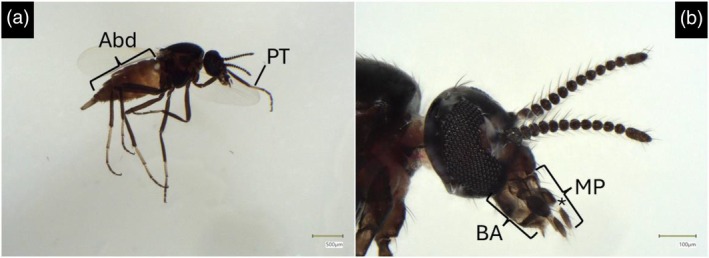
Photographs of *L. noei* (Site 1) taken under a high‐resolution stereomicroscope. (a) Whole body. The abdomen is brownish and the 1st tarsomeres of prothoracic legs are yellowish. (b) Detail of the head; the distal article of maxillary palp shows a (*) white base. Abd: Abdomen; PT: Prothoracic 1st tarsomere; MP: Maxillary palp; BA: Buccal apparatus.

The preliminary identification at sub‐genus level was made easy by the clear visualisation of the typical 14 segmented antenna of subgenus *Leptoconops*, and the absence of the deep enclosed palpal pit of subgenus *Holoconops* (Figure 2a,b). We then observed several details to identify the individuals at the species level. As shown in Figure 2, the specimens had an evident brownish colour of the abdomen, darker on the tergites and a buccal apparatus not longer than the head. Those characteristics excluded the identification of the specimens as *L. irritans*, since this species is well distinguishable by the isabelline abdomen and the proboscis, longer than the head. Once *L. irritans*, one of the most commonly found species in previous studies in Tuscany (Polidori et al., 2023 and references therein) was excluded, the use of the keys and descriptions by Clastrier and Coluzzi (1973) and Clastrier (1972) allowed the identification of all the specimens as *L. noei*. However, *L. bezzii*, *L. bidentatus* and *L. noei* are the three species of *Leptoconops* sensu stricto recorded in Italy besides *L. irritans* and are known to possess common characteristics that have historically led to confusion about their correct identification, as *L. bezzi* was the only described species in Italy prior to 1972.

**FIGURE 3:**
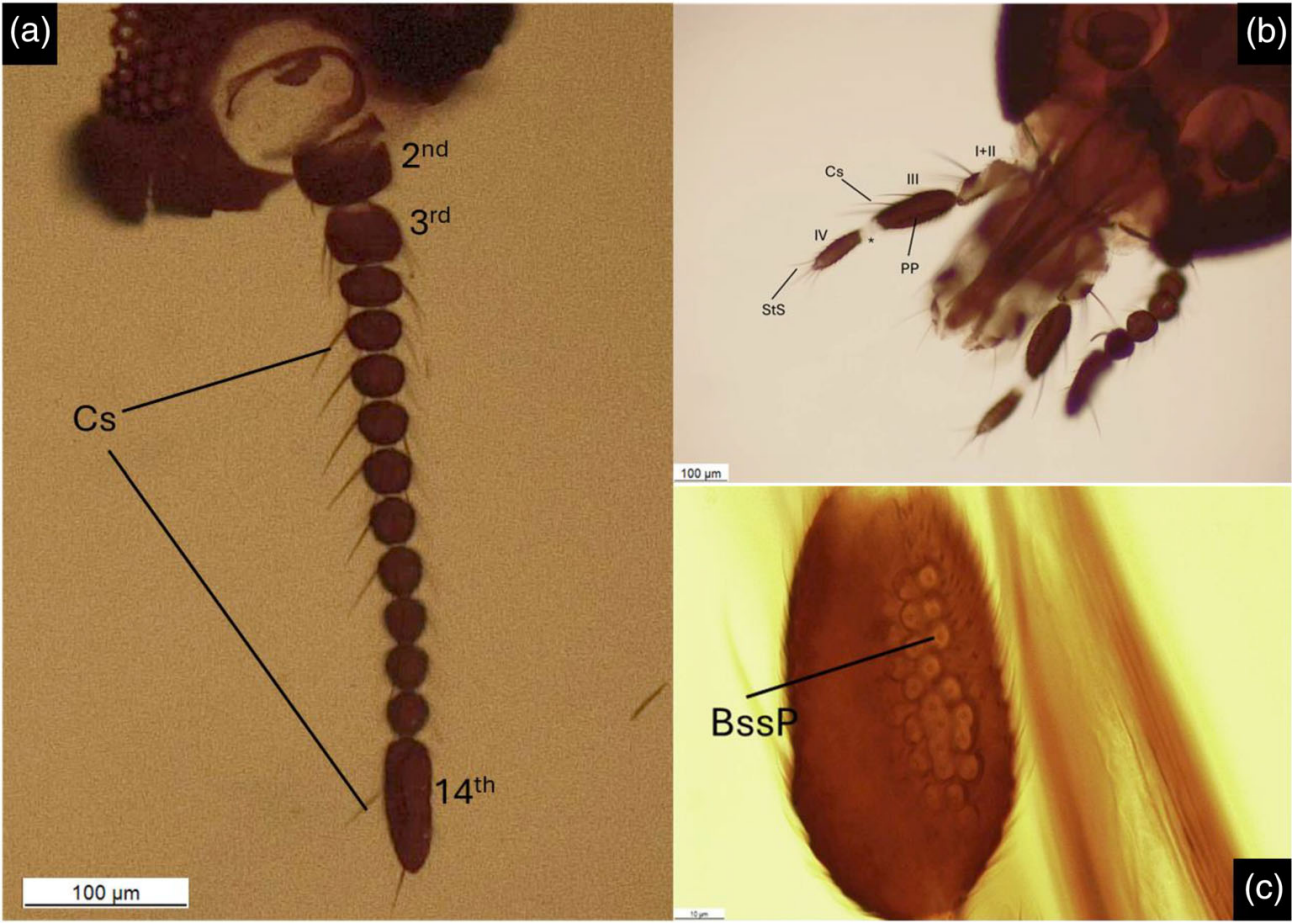
Photographs at optical microscope *L. noei* from Siena district. (a) Antenna (Site 1); 2nd, 3rd and 14th antennomeres marked. (b) Dorsal view of the head, details of maxillary palps (Site 7). I + II: 1st and 2nd palpal articles, fused; III: 3rd palpal article; IV: 4th palpal article, with (*) white base. (c) Detail of the palpal pit (Site 7). Cs: Chaetic sensilla; StS: Sharp trichoid Sensilla; BssP: Bulb‐shaped sensilla pit; PP: Palpal pit.

As reported in Figure 2, using a high‐precision digital stereomicroscope, we observed the main distinctive features of *L. noei*: the prothoracic tarsomere is yellowish, while it is brown in *L. bezzii* (Figure 2a); the 4th article of the maxillary palp is brown, except for the clear peduncle (Figures 2b and 3b), while the entire article is whitish in *L. bidentatus*; the tarsal claws present a basal tooth with a single bristle, while *L. bidentatus* has two bristles.

Observations of slide‐mounted specimens under an optical microscope allowed for the definition of some morphological details. The antenna of the samples (Figure 3a) corresponds to the description of *L. noei* and consists of 14 antennomeres, of which the 1st is thin and cup‐like, the 2nd is cylindrical and the others constitute the flagellum. The 3rd article is pear‐shaped, the 5th‐8th are round and slightly flattened, the 9th‐13th are perfectly round and the 14th is cylindrical, approximately 2.5 times as long as the 13th article.

**FIGURE 4:**
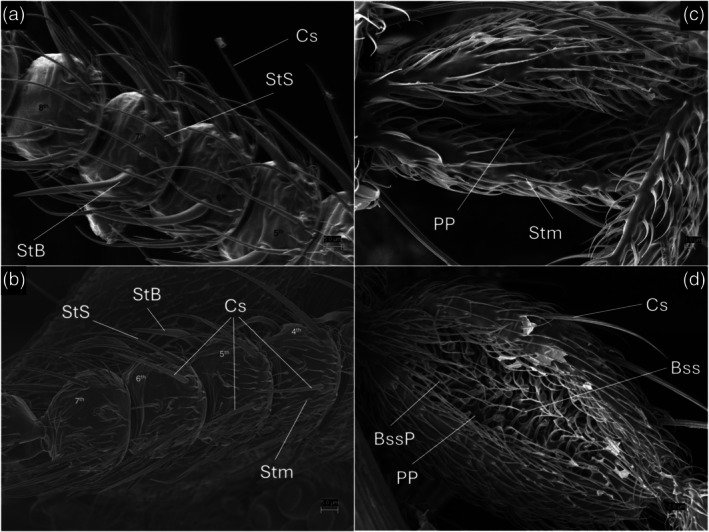
SEM photographs of *L. noei*, (a.b) Details of antennae; (c,d) Details of 3rd palpal article and palpal pit. (a) Antennomeres 5th – 8th, internal face (Site 1 specimen). (b) Antennomeres 4th – 7th, external face (Site 1 specimen). SEM photograph of 3rd palpal article and palpal pit. (c) Site 1 specimen. (d) Grosseto district specimen. Cs: Chaetic sensilla; StS: Sharp trichoid sensilla; Stm: Small microtrichoid sensilla; StB: Blunt trichoid sensilla. PP: Palpal pit; Bss: Bulb‐shaped sensilla; BssP: Pit of the Bss.

As shown in Figure 3b, the maxillary palp consists of four segments, of which the 1st and the 2nd appear fused. The 3rd segment is brown, elongated and contains a wide and shallow palpal pit (Figure 3c), while the 4th is brown except for the white base and contains 4 blunt trichoid sensilla (StB). About 40 small pits are visible inside the palpal pit, and some bulb‐shaped sensilla (Bss) emerge from its surface. However, with optical microscope observation, we found it difficult to assess the exact number of small pits inside the palpal pit and to clearly observe the Bss due to their small size and hyaline appearance. The subsequent SEM analysis permitted a better visualisation of the antennal sensilla and the structure of the maxillary palps. The observation of the sensilla on the flagellum revealed the presence of a pair of blunt trichoid sensilla (StB) on the internal side of the 3rd to 13th antennomeres, with a distinctive curved shape (Figure 4a). Three long chaetic sensilla (Cs) were observed on the external face of the 3rd to 9th antennomeres, while the 10th to 13th have just two (Figure 4b). In both cases, the Cs are twice the antennomere length, and they arise at the base of the article. The 3rd to 13th antennomeres bear crowns of sharp trichoid sensilla (StS) on their internal face, close together. These sensilla are scattered on the proximal part in groups of 6–8 and they are present on the distal part in groups of 1–3, as long as the article. The 14th article has 3 Cs and numerous StS distributed across its surface. All the articles are covered with small microtrichoid sensilla (Smt). The two SEM‐observed samples exhibited a peculiar characteristic: the palpal pits lacked any Bss and their associated circular pits. This trait sharply differentiates these specimens from *L. noei* from coastal Maremma (Figure 4d), coming from the same locality as the specimens analysed by Polidori et al. (2023): Marina di Alberese (Grosseto district, see Figure 1b). In fact, while the palpal pit surface of these two inland Tuscany specimens was smooth and covered with Stm (Figure 4c), the palpal pit of the coastal Maremma *L. noei* specimen was clearly full of Bss. Despite such a relevant difference, however, the results of molecular analyses (see below) were coherent with the identification of all examined individuals from the inland area as belonging to *L. noei*.

### Molecular phylogeny

**FIGURE 5:**
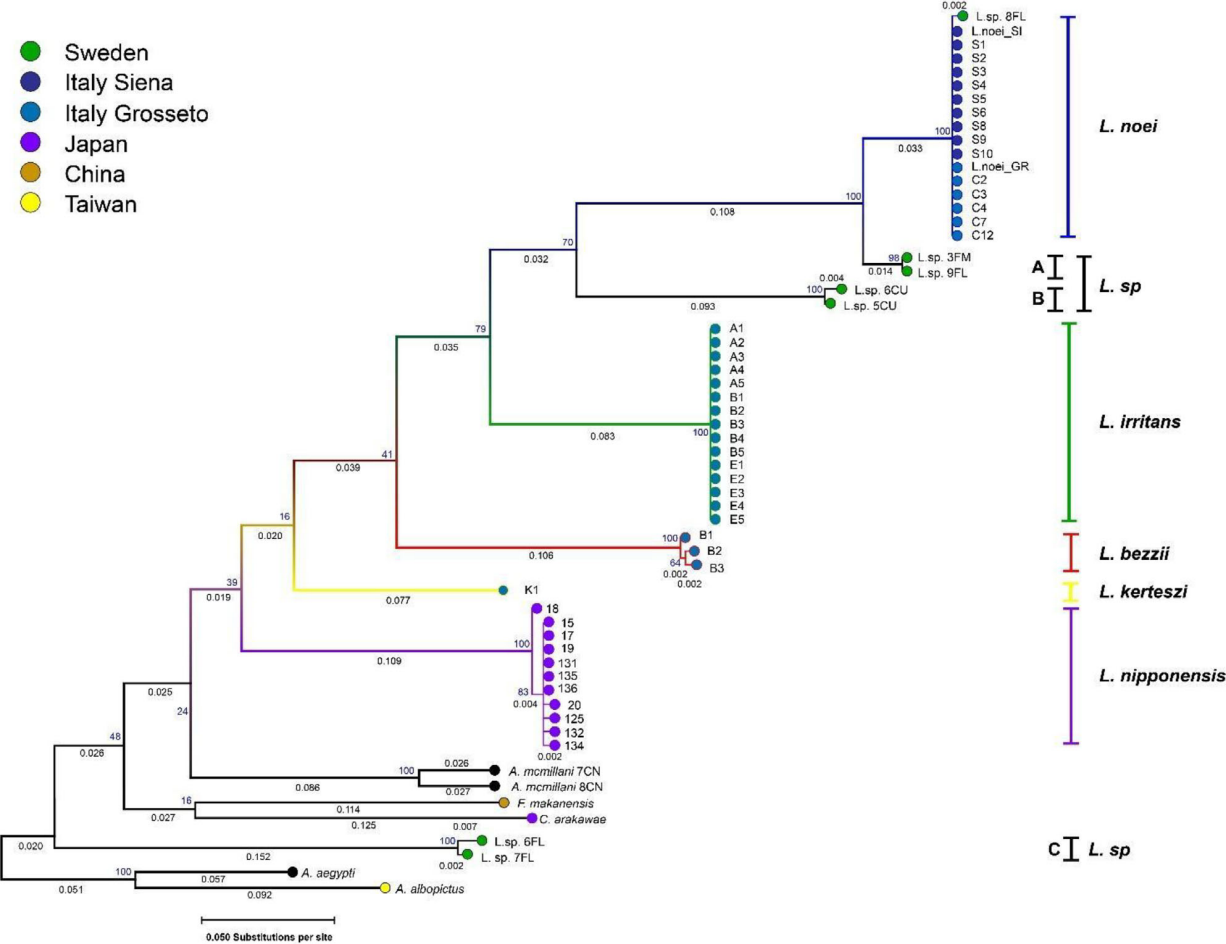
Maximum likelihood tree of all available *COX1* sequences of *Leptoconops* sensu stricto and its closest relatives published in GenBank. Branch lengths represent substitutions per site; sampling location, when known, is indicated as a coloured circle.

The phylogenetic tree built with partial *COX1* sequences highlighted the relationships between *Leptoconops* species and their closest relatives (Figure 5). As a whole, the examined *Leptoconops* spp. sequences present 173 polymorphic sites with a total number of 236 nucleotide substitutions. Average pairwise nucleotide diversity was calculated between and within species (Table S2). Variability within the genus is high, with average nucleotide diversity *π* = 14.2 ± 0.6%. *Leptoconops noei* individuals cluster in a monophyletic group without any major haplogroup; average nucleotide diversity within this species was *π* = 0.03 ± 0.02%. The same applies for *L. irritans* with the difference that no haplotype diversity is present. The first species that cluster with *L. noei* and *L. irritans*, albeit with low bootstrap support, is *L. bezzii*, presenting *π* = 0.28 ± 0.09% variability and possibly two main haplogroups. The further branch in the obtained tree is that of *L. kerteszi*. *Leptoconops nipponensis* is the most basal species of this putative *Leptoconops* clade, and also appears to have two main haplogroups, although one is found in only one individual. We included the seven entries of unclassified *Leptoconops* sp. in the attempt to clarify their taxonomy. These individuals are quite diverse and deserve a specific treatment. One individual clusters within *L. noei* individuals; two individuals form a sister group to this, with an average divergence of 4.74 ± 3.17% and are temporarily assigned to “*L. sp*. A”. Two other unclassified individuals form another monophyletic group, temporarily named “*L. sp* B”, and placed sister to *L. noei* + *L*. sp. *A. L*. sp. B exhibits levels of divergence in the range of 20% ± 1% with *L. noei*, *L. irritans* and *L. sp*. A. Finally, the last two unclassified individuals are somewhat related (1.30% divergence) and are named “*L. sp* C”. This putative new taxon clusters outside the monophyletic *Leptoconops* genus and has a rather large divergence when compared to other *Leptoconops* (~26%–31%). These two, being at the base of the Ceratopogonidae family, are therefore, more closely related to the Forcipomyiinae subfamily rather than the Leptoconopinae.

### Mitochondrial genome

We obtained the first complete mitogenome for two *L. noei* populations, one from Siena (inland, site 1) and one from Grosseto (coast); the latter was used as a reference sequence for the species.

**FIGURE 6:**
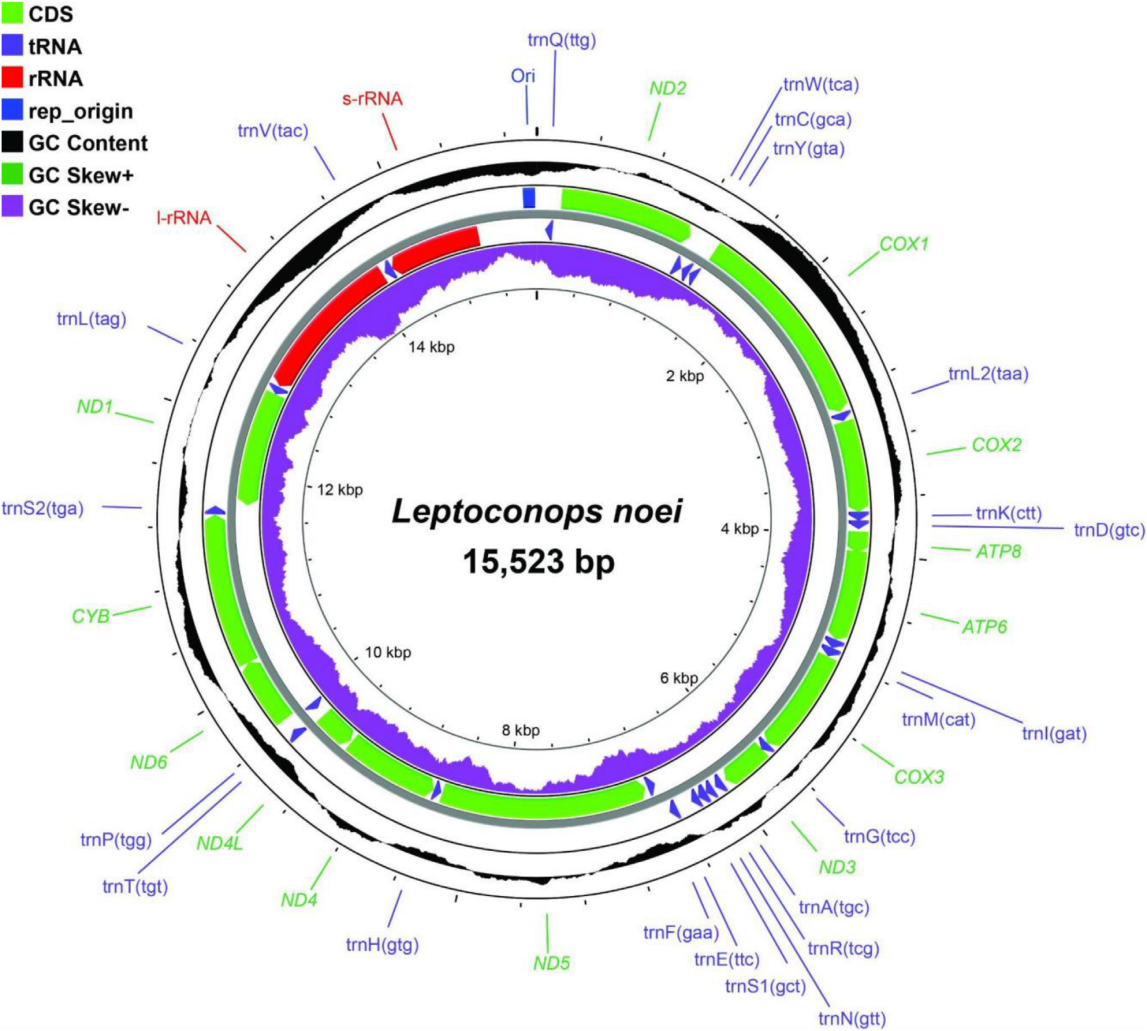
Map of *Leptoconops noei* mitogenome. This map refers to the reference sequence (*L. noei*_GR) and was obtained with the CGview Server (Grant & Stothard, 2008). Genes are represented as blocks of different colours. CDS indicates the 13 coding sequences. tRNAs are labelled according to single‐letter abbreviations. Direction of gene transcription is indicated by arrows. The GC content is plotted using a black sliding window, as the deviation from the average GC content of the entire sequence. Positive and negative GC skews are relative to the average GC content of the entire sequence.

We obtained an average of 1.1 million reads per sample with a depth of 11,000X covering the whole mitogenome. Quality scores throughout the 150 bp paired‐end reads were above Q30 at both the base and sequence level (Figure S1A,B). The gene order (Table S3) was found to be unchanged compared to the ancestral insect mitochondrial genome (Cameron, 2014). The reference mitogenome is 15,523 bp in length (Figure 6, Figure S2) and harbours 37 genes (13 protein‐coding genes, 22 tRNAs, two rRNAs) and the Control region. Gene content is identical in the other genome. We found only one polymorphic site (pos 15,222; A to G transition) and one site with a deletion (pos 6197; deletion of a nucleotide in a Poly‐A); given this, average nucleotide diversity is notably low (*π* = 0.007 ± 0.003). Sequence composition is as follows: A = 40.9% (6349 bp), T = 30.7% (4769 bp), C = 17.8% (2760 bp) and G = 10.6% (1645 bp) with low GC = 28.4%. The notable negative GC skew throughout the molecule has also been seen in other Ceratopogonidae (Beckenbach & Borkent, 2003; Gao et al., 2024). Most tRNAs displayed the characteristic cloverleaf structure except for *tRNA* ^*Cys*^, *tRNA* ^*Glu*^, *tRNA* ^*Hys*^, *tRNA* ^*Pro*^, *tRNA* ^*Arg*^ and *tRNA* ^*Trp*^ which were found to have an extended hairpin structure without the D‐ and T‐arms and *tRNA* ^*Phe*^, *tRNA* ^*Gly*^, *tRNA* ^*Ser1*^ and *tRNA* ^*Thr*^ which were missing the D‐arm (Figure S3).

## DISCUSSION

The description of new *L. noei* populations documented in this study highlights the limited knowledge of the species' distribution for this genus of biting midges. Previous investigations focused on the Maremma area, covering the coastal side of Tuscany and Lazio regions, and to a minor extent on the seaside areas of Puglia, Sardinia and Basilicata. Site S13, located in Siena municipality, is approximately 80 kilometres from the Tyrrhenian Sea and represents the most inland detection of *L. noei* ever recorded (Figure 1b). Interestingly, only González et al. (2013) reported a similar inland occurrence of several *L. noei* individuals in northern Spain. However, the absence of molecular analyses (e.g., DNA barcoding) or detailed morphological observations prevents a direct comparison between the Spanish and Italian populations. Literature on the larval habitats of *L. noei* and its closely related species *L. bezzii* (Bettini et al., 1969b; Majori et al., 1971) indicates a preference for clay‐rich soils, a characteristic feature of both the Siena and Grosseto districts. The studies conducted on the flight activity of *L. noei*, which revealed that female catching increases near the larval habitat (Carrieri et al., 2011), lead us to hypothesise that sites 1 and 2 were the nearest to the site of larval development. Collection rate is also influenced by the wind force (Carrieri et al., 2007, 2011), with a decrease in catching with stronger wind, using light traps or catching on operators, as the biting activity is negatively affected. The use of a towel, which allowed the midges to rest on it instead of being carried away, proved to be a useful tool to collect with adverse wind. Although the typical host of *L. noei* remains unknown, the presence of cattle is a key factor for the presence of other related species (Bettini et al., 1969c). This was somehow apparent in the recent study of Polidori et al. (2023), where *L. noei* was collected at locations characterised by cattle farms at more than 3 km from the sea or riverbeds, in contrast to *L. irritans*, which is more frequently collected closer to coasts. The landscapes of Siena and Grosseto are shaped by farms and pastures, but seasonal weather patterns differ between the two areas: the inland region experiences slightly colder temperatures and lower precipitation, leading to the hypothesis that *L. noei* could colonise different habitats, not necessarily similar to that of Maremma.

The Siena population was identified morphologically as *L. noei* without ambiguity, as its key features were identical to those of the reference population in Grosseto. SEM observations revealed various types and numbers of sensilla on both antennae and maxillary palps. Our analysis confirmed the pattern of the antennal sensilla as described by Clastrier and Coluzzi (1973), clearing ambiguity to avoid misidentification with *L. bezzii*. The presence of two blunt trichoid sensilla (StB) on the 3rd to 13th antennomeres and the dense crown of sharp trichoid sensilla (StS) on the same segments is hypothesised to be linked to host preferences (Isberg et al., 2013; Polidori et al., 2023). While this hypothesis requires confirmation, it is in line with observations of *L. noei* in Grosseto. In contrast, *L. irritans* has long basiconic sensilla (SbL) but no StB on most flagellomeres and fewer StS. Notably, *L. irritans* is known to cause significant nuisance due to its aggressive behaviour towards humans, particularly in coastal areas (Carter, 1921; González et al., 2025; Noè, 1907; Rioux et al., 1968). The biting behaviour of *L. noei* is reported to be less invasive on humans than *L. irritans* (Noè, 1907), as it prefers to attack livestock, and it lives near farms. Despite this, recent reports indicate occasional aggressions towards humans (González et al., 2013) and our experience is that *L. noei* does exhibit aggressive behaviour towards humans. This leads us to hypothesise a lower selectivity in host preference than in *L. irritans*, an additional trait that may favour their occurrence in a wider range of habitats.

The absence of bulb‐shaped sensilla (Bss) in the palpal pit of the two inland specimens analysed with SEM is a remarkable finding. In the Ceratopogonidae family, the palpal pit (also named sensory pit) is a region of the 3rd maxillary palp segment with a role in odour reception, thanks to the sensilla covering its surface (Rowley & Cornford, 1972). While a direct correlation between the type and abundance of sensilla and host preference remains unclear, studies on *Culicoides* spp. have shown that differences in sensilla abundance reflect varying host preferences (Braverman & Hulley, 1979; Isberg et al., 2013). Bss are characteristic of many Ceratopogonidae, present in the sensory pits of palps and antennae, and have been described in all European species of the subgenus *Leptoconops* sensu stricto. Interestingly, in coastal locations of Maremma, Bss were denser in *L. noei* than in *L. irritans*, while they are completely absent in the two inland specimens. This implies an important morphological variation among individuals and populations that may either represent different morphotypes (perhaps subspecies), local adaptations to particular environmental conditions and/or host use, or aberrant individuals. In any case, the description of this morphological variant represents a novelty in the morphological features of the genus Leptoconops that could be investigated in other populations of different areas of Italy and Europe.

Considering *COX1* sequences, there is no difference between *L. noei* from Siena and Grosseto. This is true also for *L. irritans* sampled in five locations around Grosseto, as seen in a previous work (Polidori et al., 2023). However, the presence of an individual that clusters within the *L. noei* phylogenetic group with a single nucleotide difference suggests the potential presence of some genetic variability that could be revealed through the analysis of further individuals and populations. The low genetic variability within the Italian individuals of the inland and the coastal areas likely reflects a recent founder event, possibly with ongoing genetic flow between the two areas. This is potentially facilitated by the ability of Ceratopogonidae to cover long distances by exploiting favourable winds (Jacquet et al., 2016).

This little genetic variability is further corroborated by whole mtDNA data as the Siena population has two single nucleotides polymorphisms (SNPs), Cambr differences from the Grosseto population. Moreover, *L. bezzii* and *L. kerteszi*, which had never been examined at the molecular level prior to this study, appear more basal in the *Leptoconops* species phylogeny compared to *L. noei* and *L. irritans*, with *L. bezzii* slightly closer to the two and the latter more basal. Our genetic data suggest the existence of two potentially new undescribed species (here named *Leptoconops* sp. A and B). Hypothetical species A is the sister taxon of *L. noei*, from which it is separated by a nucleotide divergence as low as 2%, a value found to differentiate species in *Culicoides* (Ander et al., 2013). Hypothetical species B shows a higher divergence compared to *L. noei* and *L. irritans*, supporting the idea that it could be a different species. In addition, according to our phylogenetic analysis, hypothetical species C could be misclassified as *Leptoconops*, as in the obtained phylogeny it is more closely related to basal Ceratopogonidae than to other *Leptoconops* species. These results are, anyway, to be interpreted with caution, considering the low bootstrap support of most of the nodes in the tree.

## CONCLUSION

In conclusion, we documented here newly identified inland populations of *L. noei* in a region of Italy previously regarded as environmentally atypical for this species. This discovery raises concerns about the possible presence of *Leptoconops* populations in other unexplored areas of Italy and Europe, including locations near urban centres. The latter being true, such proximity would pose potential risks for human exposure, leading to nuisance from biting, discomfort and in some cases allergic reactions. Furthermore, in touristic locations, infestation by *Leptoconops* may lead to non‐negligible economic damage. Finally, the description of a new morphological variation in the sensory system of *L. noei* provides valuable information for species identification. The de novo sequencing of the complete mitochondrial genome represents a milestone for the study of the genus *Leptoconops*. The annotated genes provide valuable resources across various research fields, particularly as reference markers for barcoding the genus, which still encompasses numerous ambiguously described species. Molecular identification emerges as a powerful approach to overcome the inherent limitations of morphological methods. Expanding genetic datasets beyond the conventional *COX1* marker holds significant potential for clarifying unresolved taxonomic classifications. Future genome sequencing efforts will advance our understanding of the biology of the genus *Leptoconops*, particularly in terms of metabolic pathways and pesticide resistance, providing critical insights for more effective species management in inhabited areas. We emphasise that the biology of the larval and pupal stages remains largely unexplored; further research in this domain could shed light on the distribution dynamics of *L. noei* and other species of this genus.

## AUTHOR CONTRIBUTIONS

**Giovanni Naro:** Data curation; investigation; methodology; writing – original draft. **Gianluca Lombardo:** Writing – original draft; data curation; formal analysis. **Alessandro Alvaro:** Investigation. **Laura Soresinetti:** Investigation; methodology. **Francesco Frati:** Writing – review and editing; investigation. **Luigi Marvasi:** Investigation. **Emanuele Montomoli:** Writing – review and editing; investigation. **Claudio Bandi:** Writing – review and editing; funding acquisition. **Paolo Gabrieli:** Investigation; formal analysis. **Carlo Polidori:** Formal analysis; conceptualization; writing – review and editing. **Sara Epis:** Conceptualization; investigation; funding acquisition; writing – review and editing; supervision.

## FUNDING INFORMATION

This research was supported by EU funding within the NextGenerationEU‐MUR PNRR Extended Partnership initiative on Emerging Infectious Diseases (Project no. PE00000007, INF‐ACT) (to Sara Epis and Claudio Bandi) and the National Biodiversity Future Center (Project no. CN00000033, NBFC) (to Francesco Frati). The funders had no role in study design, data collection and analysis, decision to publish or preparation of the manuscript.

## CONFLICT OF INTEREST STATEMENT

The authors declare no conflicts of interest.

## Supporting information


**Figure S1.** Sequencing quality scores: A, across all bases and B, over all sequences.
**Figure S2.** Linearised gene map of *Leptoconops noei*. Protein‐coding genes are in green, rRNA genes in red and tRNA genes in purple.
**Figure S3.** Putative secondary structure of mitochondrial tRNA genes of *Leptoconops noei*. Discriminator nucleotide is circled in red at the top of every figure and anticodon is marked with a black line at the bottom.
**Table S1.** Samples used for COX1 analysis.
**Table S2.** Average nucleotide diversity (%) within and between *Leptoconops* species/groups from different geographic areas. Average intragroup nucleotide diversities (π) are on the diagonal.
**Table S3.** Locus map of *Leptoconops noei*. * Nucleotide positions are relative to the Grosseto sample used as a reference sequence. Triplets in brackets in tRNAs are the anticodon sequences.

## Data Availability

Sequences were deposited in GenBank under accession numbers: PV056079, PV077351–PV077359 and PV081858–PV081859.
