# Supplementary material for: Discovery of a new inland population of Leptoconops noei in Italy with sequencing of the first complete mitochondrial genome for the genus
Source: Med Vet Entomol. 2025 Jul 11;39(4):817–28. doi: 10.1111/mve.12828 (PMC12586292; doi:10.1111/mve.12828)
Supplement: Supplementary file 1 — Figure S1. Sequencing quality scores: A, across all bases and B, over all sequences. Figure S2. Linearised gene map of Leptoconops noei. Protein‐coding genes are in green, rRNA genes in red and tRNA genes in purple. Figure S3. Putative secondary structure of mitochondrial tRNA genes of Leptoconops noei. Discriminator nucleotide is circled in red at the top of every figure and anticodon is marked with a black line at the bottom. Table S1. Samples used for COX1 analysis. Table S2. Average nucleotide diversity (%) within and between Leptoconops species/groups from different geographic areas. Average intragroup nucleotide diversities (π) are on the diagonal. Table S3. Locus map of Leptoconops noei. * Nucleotide positions are relative to the Grosseto sample used as a reference sequence. Triplets in brackets in tRNAs are the anticodon sequences. [file MVE-39-817-s001.docx]

**Supporting information**

**Figure S1**


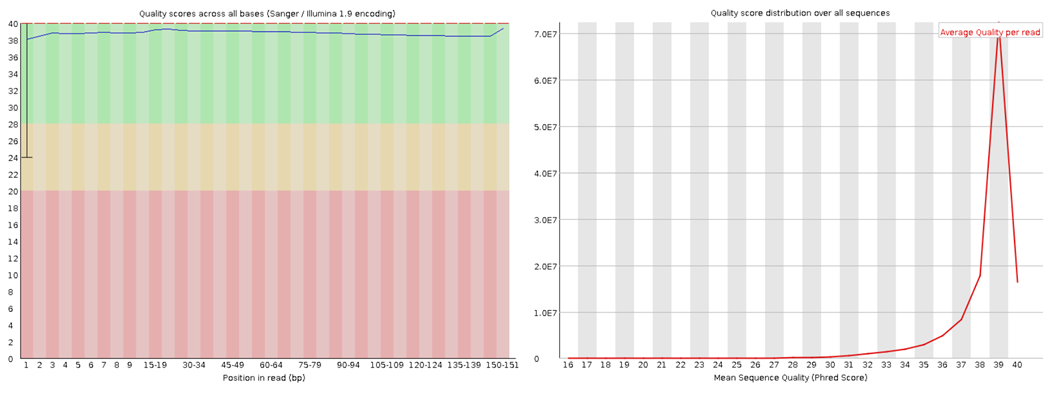


**Figure S2**

*
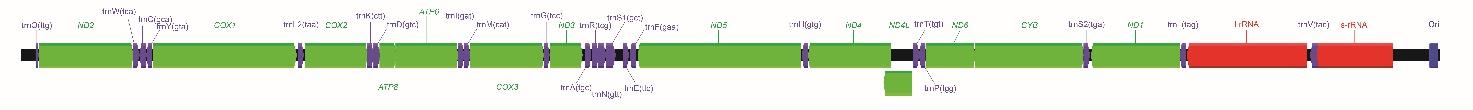
*

**Figure S3**


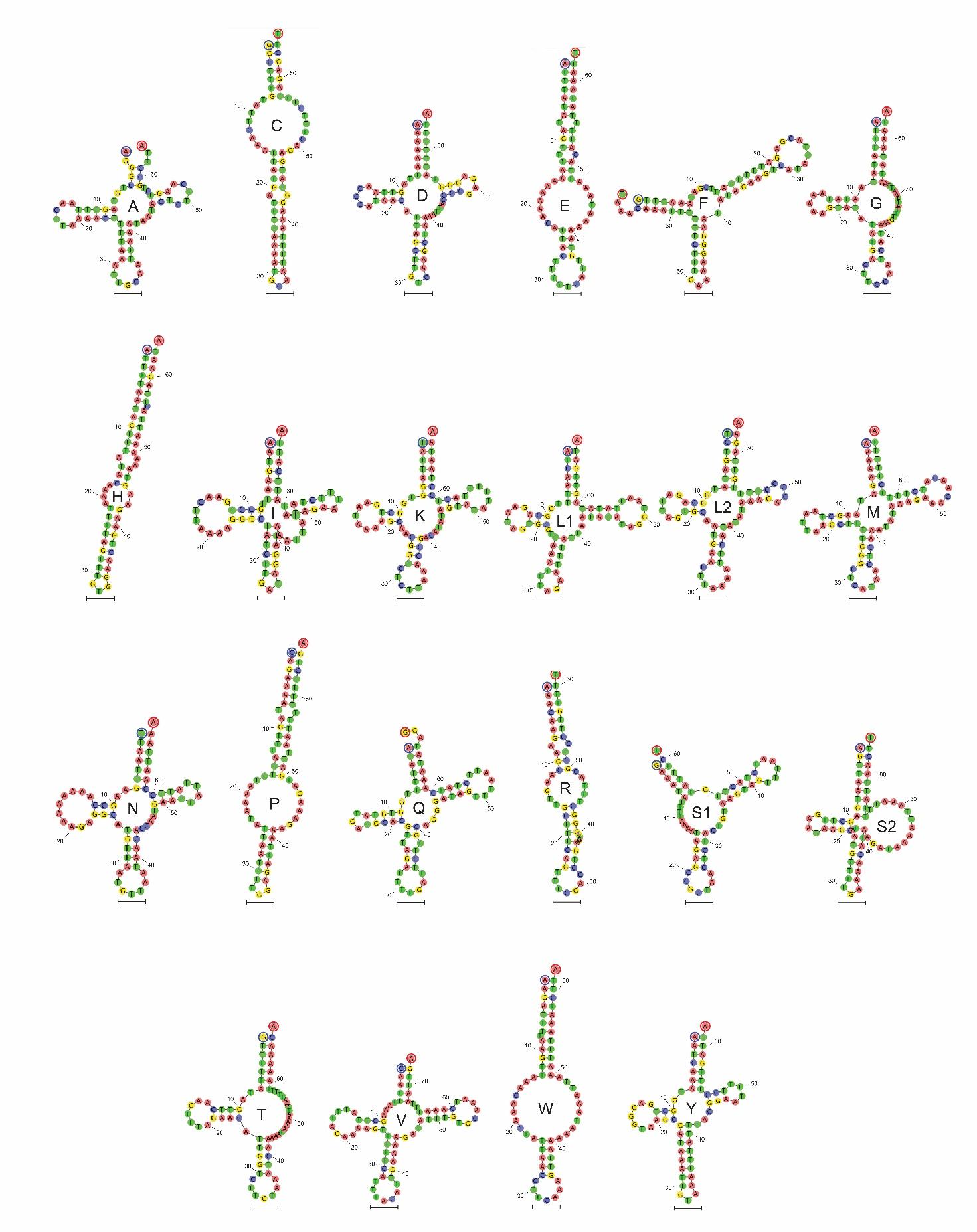


**Table S1**

| Sample name | Species | Country | Location | Accession | Work |
| --- | --- | --- | --- | --- | --- |
| A. aegypti | *Aedes aegypti* | N/A | N/A | NC_035159 | Matthews et al., 2018 |
| A. albopictus | *Aedes albopictus* | Taiwan | Taipei | NC_006817 | Ho et al., 2005 |
| A. mcmillani 7CN | *Austroconops_mcmillani* | N/A | N/A | KT278282 | Johanson et al., 2016 |
| A. mcmillani 8CN | *Austroconops_mcmillani* | N/A | N/A | KT278281 | Johanson et al., 2016 |
| C. arakawae | *Culicoides arakawae* | Japan | Okinawa | NC_009809 | Matsumoto et al., 2009 |
| F. makanensis | *Forcipomyia makanensis* | China | Guizhou | MK000395 | Jiang, et al., 2019 |
| B1 | *Leptoconops bezzii* | Italy | Grosseto | PQ789186 | This work |
| B2 | *Leptoconops bezzii* | Italy | Grosseto | PQ789187 | This work |
| B3 | *Leptoconops bezzii* | Italy | Grosseto | PQ789188 | This work |
| A1 | *Leptoconops irritans* | Italy | Grosseto | OM672396 | Polidori et al., 2023 |
| A2 | *Leptoconops irritans* | Italy | Grosseto | OM672388 | Polidori et al., 2023 |
| A3 | *Leptoconops irritans* | Italy | Grosseto | OM672395 | Polidori et al., 2023 |
| A4 | *Leptoconops irritans* | Italy | Grosseto | OM672397 | Polidori et al., 2023 |
| A5 | *Leptoconops irritans* | Italy | Grosseto | OM672390 | Polidori et al., 2023 |
| B1 | *Leptoconops irritans* | Italy | Grosseto | OM672392 | Polidori et al., 2023 |
| B2 | *Leptoconops irritans* | Italy | Grosseto | OM672386 | Polidori et al., 2023 |
| B3 | *Leptoconops irritans* | Italy | Grosseto | OM672393 | Polidori et al., 2023 |
| B4 | *Leptoconops irritans* | Italy | Grosseto | OM672394 | Polidori et al., 2023 |
| B5 | *Leptoconops irritans* | Italy | Grosseto | OM672391 | Polidori et al., 2023 |
| E1 | *Leptoconops irritans* | Italy | Grosseto | OM672398 | Polidori et al., 2023 |
| E2 | *Leptoconops irritans* | Italy | Grosseto | OM672387 | Polidori et al., 2023 |
| E3 | *Leptoconops irritans* | Italy | Grosseto | OM672385 | Polidori et al., 2023 |
| E4 | *Leptoconops irritans* | Italy | Grosseto | OM672389 | Polidori et al., 2023 |
| E5 | *Leptoconops irritans* | Italy | Grosseto | OM672384 | Polidori et al., 2023 |
| K1 | *Leptoconops kerteszi* | Italy | Grosseto | PV056079 | This work |
| 125 | *Leptoconops nipponensis* | Japan | N/A | LC662828 | Matsuba et al., 2024 |
| 131 | *Leptoconops nipponensis* | Japan | N/A | LC662823 | Matsuba et al., 2024 |
| 132 | *Leptoconops nipponensis* | Japan | N/A | LC662824 | Matsuba et al., 2024 |
| 134 | *Leptoconops nipponensis* | Japan | N/A | LC662825 | Matsuba et al., 2024 |
| 135 | *Leptoconops nipponensis* | Japan | N/A | LC662826 | Matsuba et al., 2024 |
| 136 | *Leptoconops nipponensis* | Japan | N/A | LC662827 | Matsuba et al., 2024 |
| 15 | *Leptoconops nipponensis* | Japan | N/A | LC662818 | Matsuba et al., 2024 |
| 17 | *Leptoconops nipponensis* | Japan | N/A | LC662819 | Matsuba et al., 2024 |
| 18 | *Leptoconops nipponensis* | Japan | N/A | LC662820 | Matsuba et al., 2024 |
| 19 | *Leptoconops nipponensis* | Japan | N/A | LC662821 | Matsuba et al., 2024 |
| 20 | *Leptoconops nipponensis* | Japan | N/A | LC662822 | Matsuba et al., 2024 |
| C12 | *Leptoconops noei* | Italy | Siena | OM672379 | Matsuba et al., 2024 |
| C2 | *Leptoconops noei* | Italy | Siena | OM672380 | Polidori et al., 2023 |
| C3 | *Leptoconops noei* | Italy | Siena | OM672381 | Polidori et al., 2023 |
| C4 | *Leptoconops noei* | Italy | Siena | OM672382 | Polidori et al., 2023 |
| C7 | *Leptoconops noei* | Italy | Siena | OM672383 | Polidori et al., 2023 |
| L_noei_GR | *Leptoconops noei* | Italy | Grosseto | PV081858 | This work |
| L_noei_SI | *Leptoconops noei* | Italy | Siena | PV081859 | This work |
| S1 | *Leptoconops noei* | Italy | Siena | PV077352 | This work |
| S10 | *Leptoconops noei* | Italy | Siena | PV077358 | This work |
| S2 | *Leptoconops noei* | Italy | Siena | PV077351 | This work |
| S3 | *Leptoconops noei* | Italy | Siena | PV077353 | This work |
| S4 | *Leptoconops noei* | Italy | Siena | PV077359 | This work |
| S5 | *Leptoconops noei* | Italy | Siena | PV077354 | This work |
| S6 | *Leptoconops noei* | Italy | Siena | PV077356 | This work |
| S8 | *Leptoconops noei* | Italy | Siena | PV077357 | This work |
| S9 | *Leptoconops noei* | Italy | Siena | PV077355 | This work |
| L. sp. 3FM | *Leptoconops sp.* | Sweden | N/A | KT278173 | Johanson et al., 2016 |
| L. sp. 5CU | *Leptoconops sp.* | Sweden | N/A | KT278284 | Johanson et al., 2016 |
| L. sp. 6CU | *Leptoconops sp.* | Sweden | N/A | KT278283 | Johanson et al., 2016 |
| L. sp. 6FL | *Leptoconops sp.* | Sweden | N/A | KT278180 | Johanson et al., 2016 |
| L. sp. 7FL | *Leptoconops sp.* | Sweden | N/A | KT278178 | Johanson et al., 2016 |
| L. sp. 8FL | *Leptoconops sp.* | Sweden | N/A | KT278179 | Johanson et al., 2016 |
| L. sp. 9FL | *Leptoconops sp.* | Sweden | N/A | KT278285 | Johanson et al., 2016 |

**Table S2**

| *Nucleotide diversity (%)* | *L.noei* | *L.sp. A* | *L.sp. B* | *L.irritans* | *L.bezzii* | *L.kerteszi* | *L. nipponensis* | *L.sp. C* | outgroups |
| --- | --- | --- | --- | --- | --- | --- | --- | --- | --- |
| *L.noei* (N=17) | 0.03 | 4.74 | 21.28 | 20.20 | 27.13 | 24.99 | 27.41 | 29.05 | 27.81 |
|  | ±0.02 | ±3.17 | ±14.02 | ±6.72 | ±14.67 | ±22.86 | ±8.42 | ±19.40 | ±10.83 |
| *L.sp. A* (N=2) | --- | 0.00 | 19.66 | 19.39 | 26.55 | 23.55 | 26.24 | 30.72 | 27.33 |
|  |  | ±0.00 | ±12.04 | ±13.71 | ±14.00 | ±16.65 | ±11.70 | ±18.82 | ±10.87 |
| *L.sp. B* (N=2) | --- | --- | 0.42 | 21.02 | 20.75 | 21.58 | 24.67 | 30.10 | 25.42 |
|  |  |  | ±0.21 | ±14.62 | ±9.15 | ±10.79 | ±10.38 | ±15.05 | ±8.59 |
| *L.irritans* (N=15) | --- | --- | --- | 0.00 | 19.03 | 18.58 | 18.85 | 26.45 | 23.70 |
|  |  |  |  | ±0.00 | ±10.86 | ±17.95 | ±6.12 | ±18.39 | ±9.76 |
| *L.bezzii* (N=3) | --- | --- | --- | --- | 0.28 | 20.57 | 19.23 | 26.90 | 26.34 |
|  |  |  |  |  | ±0.09 | ±9.70 | ±6.74 | ±11.86 | ±7.65 |
| *L.kerteszi* (N=1) | --- | --- | --- | --- | --- | 0.00 | 17.28 | 24.84 | 20.82 |
|  |  |  |  |  |  | ±0.00 | ±9.58 | ±12.42 | ±7.83 |
| *L.nipponensis* (N=11) | --- | --- | --- | --- | --- | --- | 0.23 | 26.01 | 23.29 |
|  |  |  |  |  |  |  | ±0.069 | ±10.94 | ±5.92 |
| *L.sp. C* (N=2) | --- | --- | --- | --- | --- | --- | --- | 1.30 | 26.33 |
|  |  |  |  |  |  |  |  | ±2.30 | ±8.78 |
| outgroups (N=6) | --- | --- | --- | --- | --- | --- | --- | --- | 22.36 |
|  |  |  |  |  |  |  |  |  | ±2.68 |

**Table S3**

| Gene name | Strand | Start (np)^*^ | End (np)^*^ | Length (bp) | Type | Description |
| --- | --- | --- | --- | --- | --- | --- |
| *trnQ(ttg)* | *-* | 72 | 140 | 69 | tRNA | tRNA glutamine |
| *ND2* | *+* | 186 | 1187 | 1002 | Gene | NADH dehydrogenase subunit 2 |
| *trnW(tca)* | *+* | 1186 | 1248 | 63 | tRNA | tRNA tryptophan |
| *trnC(gca)* | *-* | 1252 | 1316 | 65 | tRNA | tRNA cysteine |
| *trnY(gca)* | *-* | 1324 | 1385 | 62 | tRNA | tRNA tyrosine |
| *COX1* | *+* | 1398 | 2933 | 1536 | Gene | Cytochrome c oxidase subunit I |
| *trnL2(taa)* | *+* | 2947 | 3009 | 63 | tRNA | tRNA leucine2 |
| *COX2* | *+* | 3016 | 3693 | 678 | Gene | Cytochrome c oxidase subunit II |
| *trnK(ctt)* | *+* | 3695 | 3761 | 67 | tRNA | tRNA lysine |
| *trnD(gtc)* | *+* | 3759 | 3823 | 65 | tRNA | tRNA aspartic acid |
| *ATP8* | *+* | 3825 | 4001 | 177 | Gene | ATP synthase subunit 8 |
| *ATP6* | *+* | 3998 | 4666 | 669 | Gene | ATP synthase subunit 6 |
| *trnI(gat)* | *+* | 4666 | 4733 | 68 | tRNA | tRNA isoleucine |
| *trnM(cat)* | *+* | 4733 | 4801 | 69 | tRNA | tRNA methionine |
| *COX3* | *+* | 4805 | 5593 | 789 | Gene | Cytochrome c oxidase subunit III |
| *trnG(tcc)* | *+* | 5594 | 5663 | 70 | tRNA | tRNA glycine |
| *ND3* | *+* | 5663 | 6007 | 345 | Gene | NADH dehydrogenase subunit 3 |
| *trnA(tgc)* | *+* | 6035 | 6098 | 64 | tRNA | tRNA alanine |
| *trnR(tcg)* | *+* | 6118 | 6178 | 61 | tRNA | tRNA arginine |
| *trnN(gtt)* | *+* | 6178 | 6245 | 68 | tRNA | tRNA asparagine |
| *trnS1(gct)* | *+* | 6246 | 6306 | 61 | tRNA | tRNA serine1 |
| *trnE(ttc)* | *+* | 6424 | 6486 | 63 | tRNA | tRNA glutamic acid |
| *trnF(gaa)* | *-* | 6500 | 6567 | 68 | tRNA | tRNA phenylalanine |
| *ND5* | *-* | 6587 | 8293 | 1707 | Gene | NADH dehydrogenase subunit 5 |
| *trnH(gtg)* | *-* | 8303 | 8366 | 64 | tRNA | tRNA histidine |
| *ND4* | *-* | 8369 | 9712 | 1344 | Gene | NADH dehydrogenase subunit 4 |
| *ND4L* | *-* | 9706 | 9996 | 291 | Gene | NADH dehydrogenase subunit 4L |
| *trnT(tgt)* | *+* | 10,006 | 10,072 | 67 | tRNA | tRNA threonine |
| *trnP(tgg)* | *-* | 10,073 | 10,138 | 66 | tRNA | tRNA proline |
| *ND6* | *+* | 10,147 | 10,674 | 528 | Gene | NADH dehydrogenase subunit 6 |
| *cytb* | *+* | 10,674 | 11,810 | 1137 | Gene | Cytochrome b |
| *trnS2(tga)* | *+* | 11,812 | 11,875 | 64 | tRNA | tRNA serine2 |
| *ND1* | *-* | 11,890 | 12,840 | 951 | Gene | NADH dehydrogenase subunit 1 |
| *trnL1(tag)* | *-* | 12,841 | 12,907 | 67 | tRNA | tRNA leucine1 |
| *rrnL* | *-* | 12,916 | 14,183 | 1268 | rRNA | 16S ribosomal RNA |
| *trnV(tac)* | *-* | 14,206 | 14,277 | 72 | tRNA | tRNA valine |
| *rrnS* | *-* | 14,276 | 15,040 | 765 | rRNA | 12S ribosomal RNA |
| *D-loop* | *+* | 15,041 | 15,523 | 483 | CR | Control region |
| *Ori* | *+* | 15,422 | 15,510 | 89 | *ori* | Origin of replication |

**Supplementary References**

Ho,C.-M., Chang,H.-P. and Liu,Y.-M. – Unpublished work

Jiang, X., Han, X., Liu, Q., & Hou, X. (2019). The mitochondrial genome of *Forcipomyia makanensis* (Insecta: Diptera: Ceratopogonidae). *Mitochondrial DNA Part B*, *4*(1), 344-345.

Johanson, K. A. (2016). Evolutionary relationships among higher taxa of biting midges (Diptera: Ceratopogonidae) re-evaluated, based on molecular data of five protein-coding genes. In: Strandberg, J., Taking a bite out of diversity. Taxonomy and systematics of biting midges. Doctoral thesis, Stockholm University, Stockholm and Holmberg, Malmö, pp. 1–29.

Matsuba,T. and Ozuru,R. - Unpublished work

Matthews, B. J., Dudchenko, O., Kingan, S. B., Koren, S., Antoshechkin, I., Crawford, J. E., ... & Vosshall, L. B. (2018). Improved reference genome of *Aedes aegypti* informs arbovirus vector control. *Nature*, *563*(7732), 501-507.
